# The evaluation of the oxidative stress for patients receiving neoadjuvant chemoradiotherapy for locally advanced rectal cancer

**Published:** 2017

**Authors:** GL Serbanescu, MI Gruia, M Bara, RM Anghel

**Affiliations:** * “Prof. Dr. Al.Trestioreanu” Institute of Oncology, Bucharest, Romania; ** “Carol Davila’’ University of Medicine and Pharmacy, Bucharest, Romania; *** Research Department, “Prof. Dr. Al. Trestiorenu” Institute of Oncology, Bucharest, Romania

**Keywords:** rectal cancer, radiotherapy, oxidative stress

## Abstract

**Hypothesis:** Nowadays, rectal cancer is an important healthcare challenge that affects many thousands of people each year worldwide, being diagnosed especially after the age of 50 years.

**Objective:** This study attempted to evaluate the oxidative stress in patients with rectal cancer.

**Methods and results:** 30 patients from the “Prof. Dr. Al. Trestioreanu” Institute of Oncology in Bucharest were treated with neoadjuvant radiochemotherapy during 2014 and 2016 and were included in the clinical study. Blood samples were obtained in dynamics during the treatment. From the blood samples, the serum was separated and used to identify the biochemical oxidative stress parameters.

**Results:** Regarding the determination of lipid peroxides, albumin thiols, the cuprum oxidase activity of ceruloplasmin, the values registered in the dynamic of the treatment highlighted their increase to a maximum at the treatment’s endpoint due to an important oxidative stress. Regarding the serum values for total antioxidants, the results pointed out the activation of the natural protection systems, which in time were overwhelmed, due to the installed oxidative stress.

**Conclusion:** Part of the cytotoxic effect of radiotherapy was due to the production of oxidative stress. The cell was constantly exposed to the cytotoxic action of the reactive oxygen species. The obtained results indicated the dual relation to which the tumoral cell exposed itself and the installed oxidative stress, respectively, the oxidative stress being a cause or a consequence of the malign transformation.

**Abbreviations**:

CT = computed tomography, MRI = magnetic resonance imaging, ESMO = European Society for Medical Oncology, ECOG = performance status scale

## Introduction

In terms of incidence, colorectal cancer is the third most common cancer in men (10.0% of the total) and the second in women (9.2% of the total) worldwide, with an estimated 1,36 million new cases in both sexes in 2012 [**1**]. 694,000 deaths in both sexes from colorectal cancer were estimated worldwide in 2012, accounting for 8.5% of the total deaths caused by cancer [**1**]. In Europe, the incidence of rectal cancer represents 35% of all colorectal cancers (15-25/ 100000) [**2**]. In Romania, colorectal cancer is the second malignant cause of death after lung cancer [**1**]. 

Surgical intervention is the mainstay of the treatment of rectal cancers and, in 60% of the earliest stages, provides cure [**3**,**4**]. Associated to surgery, pelvic radiation represents a standard procedure in the management of locally advanced rectal cancers [**2**,**5**]. In combination with 5-FU based chemotherapy, radiotherapy increases the sphincter preservation rate in the neoadjuvant setting and decreases local relapse rates [**6**].

The antineoplastic effect of radiotherapy is due to the production of oxidative stress and can result through two mechanisms: it leads to the damaging of the cellular macromolecules (lipids, proteins, DNA) and indirectly by determining abnormal signaling at cell level [**7**].

## Materials and Methods

30 patients who were treated in “Prof. Dr. Al. Trestioreanu” Institute of Oncology in Bucharest were enrolled in this study between January 2014 and September 2016.

The inclusion criteria were: age over 18 years, histopathological confirmation of rectal cancer, ECOG ≤ 2 performance status, no distant metastasis, no prior pelvic radiation, informed consent signed by all the patients and approved by the Ethics Committee of the Institute of Oncology. Pre-therapeutic evaluation consisted of an initial physical and rectal examination, complete blood count, liver and renal function assessment, CEA level, chest X-ray/ CT and abdominal and pelvic CT/ MRI. 

The treatment scheme consisted in concomitant radiochemotherapy due to a locally advanced tumor (T3 or T4) or lymph node involvement suspicion (N+), according to ESMO international guidelines. All the patients underwent external beam radiation therapy up to a total dose of 50-54 Gy, in daily 180-200 cGy fractions, 5 days per week. Chemotherapy schedule was based on Capecitabine 825mg/ m2 twice a day, at 30 min after main meals, 5 days per week, during radiotherapy. 6 weeks after the end of the oncological treatment, the patients were evaluated for surgery.

Blood samples were obtained in dynamics during radiotherapy as it follows: at the initiation of the treatment, at the 1/ 3 and 2/ 3 of the total radiation dose and at the end of radiotherapy. From the blood samples (5ml each of them), the serum was separated by centrifugation and used to identify the biochemical oxidative stress parameters such as: 

Malondialdehyde, the final result of the lipid peroxidation reaction, was determined by using the Carbonneau method. It had normal levels between 0-2 μmol/ 100 ml serum;

Serum ceruloplasmin is an acute phase protein. The liver is the most important site where the synthesis of ceruloplasmin takes place, but it can also occur extrahepatically. The Ravin method was used for its measurement, which was based on its p-phenylenediamine oxidase activity. Normal values were registered between 80 and 120 U.I.;

Albumin thiol compounds, part of the serum antioxidants, were determined with the Albini method. This test is based on the capacity of the SH groups to develop a colored complex with the acid 5,5-dithio-bis-2-nitrobenzoic. Normal levels were encountered among 370 and 450 μmol/ l.

The serum’s capacity to reduce iron through the redox colorimetric test with reductants was used to determine the total antioxidants. Normal values were between 0,9 and 1,4 μmol/ l. 

## Results

**Fig. 1:**
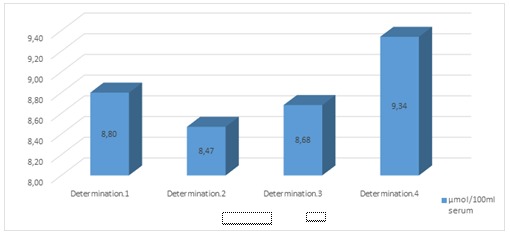
Determination of the lipid peroxidation reaction (μmol/ 100 ml serum)

It is known that the lipids, which are components of the cell membrane, represent the primary target of the oxidative attack [**8**]. The results showed an overproduction of oxidants, for example free radicals. Therefore, the lipid peroxidation reaction started and presented the maximum values at the end of radiotherapy, due to the installation of the oxidative stress. The result of the second determination was smaller than the initial one, probably, due to antioxidant endogenous systems that were activated as an adaptive mechanism.

**Fig. 2:**
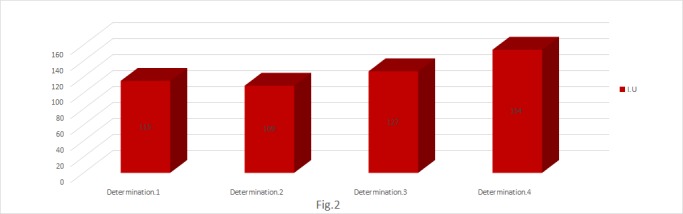
Determination of ceruloplasmin (I.U.)

To confirm the installation of an important oxidative stress, the determination of the activity of serum ceruloplasmin was introduced in the study. 

The increase of the recorded values in the dynamics of radiotherapy was significant and associated with the induction of an irreversible and intense oxidative stress. The obtained values were in accordance with those from the determination of the lipid peroxidation.

**Fig. 3:**
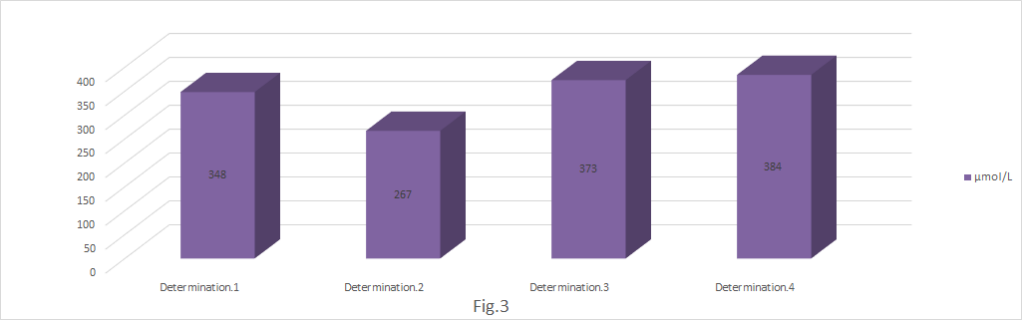
Determination of thiol compounds (μmol/ l)

The thiol groups were part of the serum antioxidant defense system by having the capacity to cancel the oxidative reactions through the inactivation of the alkoxy and hydroxyl radicals [**9**]. By own oxidation, the thiol compounds interposed to the aggressive reaction of other free radicals. However, the obtained values were a consequence of the oxidative degradation of the serum albumins. Proteins represented the second target for the oxidative stress, suggesting the fact that the oxidative stress was a continuous, irreversible process. The profile of the graphic showed a directly proportional relation between the doze of irradiation and the formation of the free thiol compounds. The value of the second determination remained low, apparently unexplainable, but we believed that it was given by the recovery mechanisms.

**Fig. 4:**
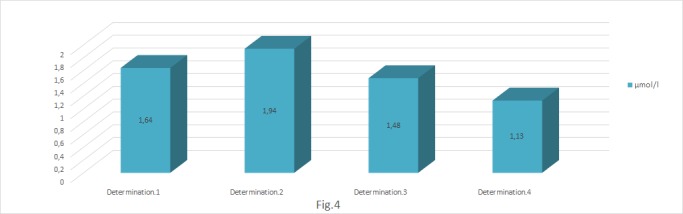
Determination of total antioxidant level (μmol/ l)

As a response to the installed oxidative stress, initially an increase of the endogenous antioxidant activity, which decreased in time, was recorded. The results pointed out the activation of the natural protection systems, which were overwhelmed in time due to the installed oxidative stress.

## Discussion

Many complex molecular and cellular alterations are involved in the process of chemical carcinogenesis [**10**,**11**]. The results of a normal cellular metabolism are the reactive species (such as oxygen reactive species), formed especially by the mitochondrial respiratory chain [**12**,**13**]. It has been demonstrated that nitric oxide can be generated also in the mitochondrial respiratory chain, but under hypoxic conditions; it can form other reactive species such as malondialdehyde [**12**]. One of the most important actions of reactive species is to determine damage to DNA, which is not sufficient to produce cancer and they may also play an important role in promoting proliferation, tumor invasion and development of metastases [**14**]. Once formed, they are rapidly decomposed by the cellular enzymatic or non-enzymatic systems. The state of oxidative stress appears when these systems are overwhelmed by the in excess formation of the reactive species through an endogenous mechanism or exogenous factors [**10**,**12**,**15**].

Oxidative and antioxidative processes determine an electron transfer, this being the reason for the cellular redox imbalance, which represents a characteristic of cancer cells [**15**,**16**].

Even if oxidative stress is involved in the carcinogenesis, there is no sufficient data of the association between rectal cancer risk and parameters of oxidative stress [**17**].

Our study highlighted that an oxidative stress was installed during radiochemotherapy. We also pointed the fact that at the beginning of radiochemotherapy all serum levels of the oxidative stress parameters (malondialdehyde, serum ceruloplasmin and albumin thiols) were higher than the normal ones, suggesting that their high pre-therapeutic serum level could be associated with an increased risk of rectal cancer.

Preoperative radiochemotherapy is established as a standard treatment for locally advanced rectal cancer based on the result of the CAO/ARO/AIO94 trial, which demonstrated a better local control after neoadjuvant versus adjuvant radiochemotherapy [**18**]. In addition, in an editorial, Glimelius recalled that concomitant radiochemotherapy decreases the risk of local relapse with 60-65% in the neoadjuvant setting compared to 20-40% when administered postoperative [**19**]. This difference was probably related to a restocking with tumor cells in the period between surgery and the initiation of radiotherapy or to the increase in free radicals determined by the surgical intervention [**19**,**20**]. Other explanation can be the hypoxic status of the cancer cells localized in the surgical bed, because it is known that hypoxia is the most important cause of radioresistance and local treatment failure [**21**].

## Conclusion

The cell is constantly exposed to the cytotoxic action of the reactive species. The cancer cell is the subject of a dual cause-effect relationship and that is the reason why we cannot say with absolute certainty if oxidative stress is a cause or a result of a malignant transformation at this level.

Many in vivo and in vitro studies demonstrated that compared to normal ones, cancer cells are exposed to an intense, continuously, sublethal oxidative stress.

**Source of founding**

This work received financial support through the project entitled “CERO – Career profile: Romanian Researcher”, grant number POSDRU/159/1.5/S/135760, co-financed by the European Social Fund for Sectoral Operational Programme Human Resources Development 2007-2013.

**Disclosures**

Authors declare that there is no conflict of interest regarding the publication of this paper.
